# Transdiagnostic Treatment of Co-occurrence of Anxiety and Depressive Disorders based on Repetitive Negative Thinking: A Case Series

**Published:** 2015-06

**Authors:** Mehdi Akbari, Rasool Roshan, Amir Shabani, Ladan Fata, Mohamad Reza Shairi, Firouzeh Zarghami

**Affiliations:** 1 Kharazmi University, Tehran, Iran; 2 Shahed University, Tehran, Iran; 3 Iran University of Medical Sciences, Tehran, Iran

**Keywords:** *Transdiagnostic Treatment*, *Repetitive Negative Thinking*, *Anxiety*, *Depression*.

## Abstract

**Objective:** The transdiagnostic cognitive behavioral treatments for treating the coexistence of anxiety and mood disorders received useful empirical supports in the recent years. However, these treatments still have moderate efficacy. Following the improvements and developments in transdiagnostic protocols and considering the importance of repetitive negative thinking as a core transdiagnostic factor in emotional disorders, this study examined a new form of transdiagnostic treatment based on Repetitive Negative Thinking (TTRNT) of co-occurrence of anxiety and depressive disorders.

**Methods:** Treatment efficacy was assessed using single case series with multiple baselines. Three patients meeting the criteria for co-occurrence of anxiety and depressive disorders were selected using the Anxiety Disorders Interview Schedule for DSM-IV. The patients were treated individually for 12 weekly sessions. Participants completed the standardized outcome measures during the baseline, treatment and one-month follow-up.

**Results:** At post-treatment, all participants showed significant clinical changes on a range of standardized outcome measures, and these gains were largely maintained through the one-month follow-up both in the principle and co-principal diagnosis.

**Conclusions:** Although the results of this preliminary investigation indicated that TTRNT could be a time effective and efficient treatment for individuals with co-occurrence of anxiety and depressive disorders, further controlled clinical trials are necessary to examine this new treatment approach.

## Introduction

Psychological treatments. One of the main problems faced by specific cognitive-behavioral therapies is the comorbidity of anxiety and mood disorders (13).

Due to complications caused by comorbidity, using this specific therapeutic protocol is not efficient for several reasons. First, high level of comorbidity of anxiety and mood disorders provokes patients’ withdrawal during specific cognitive behavioral therapies and reduces patient compliance to finish their treatment. Second, comorbidity of anxiety and mood disorders lowers the efficacy of specific cognitive behavioral therapies since using several therapeutic protocols for patients with co-occurrence of anxiety and mood disorders is not economically efficient, and these patients cannot afford to finish their treatment. Third, coexistence of anxiety and mood disorders raises severe dangers including risk of suicide in patients and requires serious interventions (14-16).

Approaching comorbidity of anxiety and mood disorders and resolving previously mentioned challenges is only possible by developing methods that consider comorbidity at both theoretical and practical levels. Transdiagnostic therapies are leading in this new approach toward development of therapeutic protocols. Transdiagnostic approaches are trying to resolve therapeutic problems and challenges arising from comorbidity by studying theoretical nature of comorbidity and recognizing common aspects of emotional disorders particularly anxiety and depression disorders (17-21; 15, 16).

Based on the review article on transdiagnostic therapies, early published transdiagnostic therapies were pragmatic. These therapies were the first attempts to treat comorbidity anxiety and mood disorders. These protocols are mainly based on clinical experiences and common and similar techniques of specific cognitive behavioral therapies and concentrate on common symptoms of anxiety and depression disorders. Several studies have shown the effect of pragmatic transdiagnostic therapies in treating emotional disorders. However, results of most of transdiagnostic therapies implicate the moderate effectiveness of these therapeutic protocols. Based on this meta-analysis, the most important justification for the moderate effectiveness of pragmatic transdiagnostic protocols is its disregard for common cognitive-behavioral mechanisms of emotional disorders (16, 18, 19, 22, 23). 

Moderate effectiveness of pragmatic transdiagnostic therapies led to designing protocols that had other foundations. Therefore, Barlow et al. (24) made a considerable improvement in designing theoretical-practical transdiagnostic protocols through several studies with emphasis on the role of ‘emotion regulation’ as the main cognitive-behavioral mechanism in emotional disorders. This protocol is more effective than other pragmatic transdiagnostic therapeutic protocols since it has a greater theoretical basis. Although in Barlow’s model, theory and therapy come together and ‘emotion regulation’ is considered and emphasized in the context of treatment, yet it does not sufficiently match with the theoretical knowledge of common psychopathology of emotional disorders (24-26).

Recent studies have shown that repetitive negative thinking is the most important common cognitive behavioral process in formation and continuation of emotional disorders. Repetitive negative thinking includes worry, rumination, treatment monitoring, obsessions and any other kind of repetitive negative thoughts which is the core of depression and anxiety disorders. Surveying more than 50 studies in cognitive pathology of emotional disorders shows that repetitive negative thinking is the main transdiagnostic factor in more than 13 diagnostic classes, including depression disorders, anxiety disorders, sleep disorder, eating disorder, substance abuse disorder and schizophrenic disorder (17-32).

Considering these studies, One potential way to improve the efficacy of pragmatic transdiagnostic therapies and Barlow’s transdiagnostic therapy (24) for co-occurrence of anxiety and depressive disorders is to adapt transdiagnostic therapies to specifically address repetitive negative thinking as core transdiagnostic processes underlying emotional disorders. One of the possible deficits of this is their disregard for repetitive negative thinking as the core process underlying emotional disorders in the context of their therapeutic protocols. Following the improvements and developments in transdiagnostic protocols and considering the importance of repetitive negative thinking in formation and continuation of emotional disorders and considering the lack of a transdiagnostic protocol based on this fundamental process, Akbari et al. (33) attempted to develop an initial design of a transdiagnostic treatment based on repetitive negative thinking (TTRNT) to be used for patients with co-occurrence of anxiety and depressive disorders. Thus, the aim of the current study was to use a single case series to establish the preliminary evidence of whether TTRNT has the potential to be an efficacious therapy for co-occurrence of anxiety and depressive disorders.

## Material and Methods

*Design*

This case series employed an AB replication across patients design with follow-up to evaluate the effectiveness of TTRNT for people with co-occurrence of anxiety and depressive disorders. Replication across three patients with different co-occurrence of anxiety and depressive disorders presentations constitutes a ‘direct replication’ (34) and begins to establish the generalizability of treatment efficacy across the disorder. This is especially important in co-occurrence of anxiety and depressive disorders as the clinical presentation which is markedly heterogeneous. Patients were assigned into no-treatment baselines for three weeks until stability was established at baseline. Patients met with the therapist on a weekly basis for 15 minutes during the baseline to complete outcome measures. No therapeutic input occurred during these meetings. Following the baseline period, TTRNT was delivered weekly, with each treatment sessions lasting no longer than one hour. Following the treatment, patients were followed up in one month; no additional treatment was delivered during the follow-up period.

*Patients*

Three patients, who consecutively referred to Counseling and Psychological Services Center of Sharif University to receive treatment for co-occurrence of anxiety and depressive disorders, were included in the study. Patients initially completed a written informed consent, and were then assessed using The Anxiety Disorders Interview Schedule for DSM-IV (ADIS-IV) and were included in the study if they met the following inclusion criteria: Having the diagnostic criteria of more than one for co-occurrence of anxiety and depressive disorders including generalized anxiety disorder, obsessive compulsive disorder, panic disorder, social anxiety, dysthymic and major depressive disorder for at least one year; aged 18–65; patient consent to participate in the research; medication free or stable on medication (i.e., three months without a change in medication type and dose). The exclusion criteria were as follows: The evidence of a psychotic disorder, bipolar disorders, substance abuse; receiving concurrent psychological treatment; having cognitive behavior therapy, behavior therapy or cognitive therapy in the two years preceding referral; and evidence of a serious problem during the study such as suicidal thoughts or change in rates of psychotropic medications dose. 

*Instrument*

Anxiety Disorders Interview Schedule for DSM-IV (ADIS-IV; 35)

The ADIS-IV is a structured diagnostic interview designed to assess the presence, nature, and severity of DSM-IV anxiety, mood, and somatoform and substance use disorders. Brown, DiNardo, Lehman, and Campbell (35) provided evidence of acceptable inter-rater reliability for the anxiety disorders investigated in the present study (k = .59 – .79). Inter-rater reliability (k = .63) for the combined depressive disorders group (major depressive disorder and dysthymic) was also acceptable. Evidence of construct validity, including discriminant and convergent validity, has been demonstrated (35). Principal and additional diagnoses are assigned a clinical severity rating (CSR) on a scale from 0 (no symptoms) to 8 (extremely severe symptoms), with a rating of 4 or above (definitely disturbing/disabling) passing the clinical threshold for DSM-IV diagnostic criteria. This measure has demonstrated excellent to acceptable inter-rater reliability for the anxiety and mood disorders (35). The full ADIS-IV-L (focusing on current and lifetime diagnoses) was administered only at the original intake. An abbreviated version of the ADIS, focusing only on current symptomatology (Mini-ADIS-IV; 35) was administered at post-treatment and follow-up. This measure has demonstrated acceptable to excellent inter-rater reliability for the anxiety and mood disorders in Iranian samples (36).

*Beck Depression Inventory-II (BDI-II;* 37 *)*

The BDI-II is a 21-item measure to assess current depressive symptoms, and was included as a general measure of depressive symptoms across the disorders. Internal consistency (α = .92) and test re-test reliability (r = .93 over 1 week) are established (37), and evidence for construct validity has been demonstrated (38). Psychometric features of revised form of this questionnaire have been obtained by Ghasemzadeh, et al. (39) in Iran. The results indicate excellent internal consistency (α = .87) and acceptable test-retest reliability (r = .74 over 1 week)

*Beck Anxiety Inventory (BAI;* 40 *)*

The BAI was included as a general measure of anxiety-related symptoms across the disorders. The BAI also contains 21 items scored in a similar way and focuses on common symptoms that are more unique to anxiety, such as somatic and certain cognitive symptoms. The reliability and validity of the BAI Farsi translated version was demonstrated by Fata et al. (41) in Iranian samples. The results indicate excellent internal consistency (α = .92) and good temporal stability (r = .81 over 1 week).

*.* *Positive and Negative Affect Scale (PANAS;* 42 *)*

The PANAS was included to assess the levels of positive and negative affect across the disorders. The PANAS is a brief, reliable, and valid self-report measure of positive and negative affect. It consists of 20 feeling or emotion words. Respondents rate each emotion word on a scale ranging from 1 (very slightly or not at all) to 5 (extremely), indicating the extent to which they experienced that emotion or feeling during the past few weeks. The PANAS allows for the assessment of core negative affect as well as deficits in positive affect. The PANAS has shown excellent convergent and divergent validity. The reliability and validity of the PANAS Farsi translated version was demonstrated by Bakhshipour (43) in Iranian samples. He showed that alpha coefficient was .87. The results indicate PANAS has construct validity.

*Repetitive Thinking Questionnaire (RNQ;* 44 *)*

The RTQ is a 31-item self-report measure of transdiagnostic repetitive negative thinking. Items were drawn from the Penn State Worry Questionnaire (45), the Ruminative Responses Scale of the Response Styles Questionnaire (46) and the Post-Event Processing Questionnaire-Revised (47) and were modified to remove diagnosis-specific content. The questionnaire comprises two subscales; Repetitive Negative Thinking (27 items) and Absence of Repetitive Thinking (4 items). This tool demonstrated the factor structure, internal consistency (α = .72–.93), convergent validity, and predictive utility of the RTQ in a student sample (44). In Iran, Khaleghi et al. (48) has reported the internal consistency of 0.89. Similarly, the rates of correlation for BAI and BDI-II were .53 and .52, respectively.

*Work and Social Adjustment Scale (WSAS;* 49 *)*

The WSAS is a 5-item measure asking participants to rate the degree of interference caused by their symptoms in work, home management, private leisure, social leisure, and family relationships. Interference is rated over the past week on a 0 to 8 scale (0 = not at all interfering to 8 = severe interference). The WSAS is a descriptive measure of subjective interference in various domains of living. The final score represents the average of scores across domains. The WSAS has shown adequate internal consistency, ranging from 0.70 to 0.94, and test-retest correlation (0.73) in a clinical sample (50). Psychometric features of revised form of this questionnaire have been obtained by Solimani, et al. (51) in Iran. The results indicate acceptable test-retest reliability (r = .69 over 1 week). Similarly, the rates of correlation for WSAS and the Depression, Anxiety and Stress Scales (DASS) were .66. (51)

*Yale-Brown Obsessive Compulsive Scale (Y-BOCS;* 52)

The Y-BOCS is a semi-structured interview designed to measure the severity of OCD symptoms. The severity scale is comprised of 10 items, 5 items measuring the severity of obsessions and 5 measuring the severity of compulsions. Each item is rated on a 5 point scale ranging from 0 (none) to 4 (extreme) giving a maximum total score of 40. The Y-BOCS has become the ‘gold standard’ assessment measure in treatment outcome research in OCD. It has been shown to have reasonable psychometric properties and sensitivity to treatment effects (53, 54). The reliability and validity of the BDD-YBOCS Farsi translated version was demonstrated by Rabiei, Khormdel, Kalantari, and Molavi (55) in both healthy and clinical samples. They showed that alpha coefficients ranged from .78 to .93 for the BDD-YBOCS total score and for its subscales.

Penn-State Worry Questionnaire (PSWQ; 45)

The PSWQ is a widely used 16-item measure of worry with excellent internal consistency (α = .86–.95) and good temporal stability (r = .92 over 8–10 weeks and r = .74–.93 over 4 weeks; 45, 56). The measure has demonstrated the evidence of construct validity in clinical and community populations (57, 58). The PSWQ has become the ‘gold standard’ assessment measure in treatment outcome research in GAD. It has been shown to have reasonable psychometric properties and is sensitive to treatment effects (59, 60). Psychometric features of this questionnaire have been obtained by Shirinzade, et al. (61) in Iran. The results indicate excellent internal consistency (α = .86) and good temporal stability (r = .90 over 4 weeks).

*Procedure*

Patients completed the BAI, BDI and WSAS at all baseline and treatment sessions and at follow-up. The PANAS, PSWQ, Y-BOCS and RNTQ were completed at the first baseline session, post treatment and 1-month follow-up. All patients would be offered 12 one hour sessions of TTRNT. After the baseline phase, patients received treatment. In the current study, assessment of the tools used in research was performed by a trained assessor. The assessor was not permitted to act as a therapist for the same case, but acted as an assessor at post-treatment and follow-up. The assessor was blind to the study. 

*Treatment*

The transdiagnostic therapy based on repetitive negative thinking (33) is a flexible, modular-based individual treatment protocol. The procedure of the protocol design in summary is as follows: Akbari et al. (33) first, incorporated repetitive negative thinking and its relevant variables into Barlow’s transdiagnostic protocol and then consulted with national and international experts in transdiagnostic therapies and at the end, redesigned Barlow’s transdiagnostic protocol based on repetitive negative thinking. The treatment protocol was approved by an institutional review board. Finally, this protocol was designed in six modules that usually take 12 one-hour sessions to administer. Therapeutic sessions are held weekly. Similar to most of the cognitive behavioral therapeutic protocols, sessions are started by reviewing previous homework. After reviewing, basic and doable concepts are presented in the session in order for the patient to be familiar with therapeutic skills. Direct training and cooperative empiricism constitute the main part of the therapeutic sessions. In the end of each session, the homework that should be done for the next session is discussed. Each module of therapy usually takes 2 sessions and some modules are emphasized based on the type of the disorder. In the following, a brief description of each therapeutic module is presented. 

 The therapist for the study was a doctoral student with 4 years of experience, who provided treatment under the close supervision of a licensed senior team member. Treatment adherence was monitored during weekly supervision and manual development meetings. Sessions were audio-taped to facilitate supervision and monitor adherence.

*Module 1: Familiarity with Emotional Disorders and Transdiagnostic Therapy*

The main purpose of this module is the client’s general awareness of emotional disorders and the necessity of utilizing transdiagnostic therapy for his/her co-occurrence of emotional disorders (24). The main part consists of familiarizing the client with treatment procedure, nature of negative and unpleasant emotions and their destructive effect on people’s performance. During this module, the patient is expected to gain more consciousness about the symptoms of his/her emotional disorders and their simultaneity and understand the essential role of the transdiagnostic therapy for his/her co-occurrence disorder.

Module 2: Emotional Self-Awareness and Emotional Mindfulness

General objectives of this module include teaching emotional self-awareness and emotional mindfulness skills (24). Content of the first objective is psychological training in nature of emotions, main components of emotional experience in order for the patient to gain a better understanding of his/her negative emotions and emotional response patterns by searching and monitoring his/her emotional experiences. Content of the second objective of this module is the emotional mindfulness skill which means that the patient can experience his/her negative emotions without judgment or inhibition. In this module of therapy, the therapist reminds the client that emotions are similar to sea waves as they come, soar and plummet so he/she could identify their presence and let these emotions go. 

Module 3: Familiarity with Repetitive Negative Thinking and Guidelines to Decrease It 

The main objectives of this module include familiarity with repetitive negative thinking, practicing attention-training technique (62) and practicing detached mindfulness technique (62) in order to reduce repetitive negative thinking. In this module, first, the therapist tries to familiarize the patient with repetitive negative thinking as the main transdiagnostic element in anxiety and depression disorders and describes this mechanism in concept of rumination, worry, preoccupation, fixation of attention on threat, self-monitoring and any other sort of repetitive negative thinking. After describing the concept of repetitive thinking and its role in emotional disorders, the therapist teaches techniques of attention training and detached mindfulness to the patient for reducing repetitive and stubborn thoughts. During this module, the therapist attempts to improve the flexibility of patient’s attention and release him/her from repetitive negative thinking using these two techniques.

Module 4: Appraisal Metacognitive Beliefs and Reappraisal

The main objectives of this module include patient’s familiarity with metacognitive beliefs about repetitive negative thinking and challenging these beliefs (62). At first, the therapist tries to inform the patient about negative and positive metacognitive beliefs related to repetitive negative thinking and identify them with the patient’s help. Then, the therapist challenges negative metacognitive beliefs in two areas of uncontrollability and repetitive negative thinking, employing verbal reattribution and behavioral experiments techniques. Finally, after modifying negative metacognitive beliefs, the therapist makes an attempt to correct positive metacognitive beliefs related to repetitive negative thinking and tries to challenge and renovate these believes using employing verbal reattribution and Behavioral experiments techniques.

*Module 5: Facing Experiential Avoidance*

The main objectives of this module include patient’s familiarity with the concept of experiential avoidance and training in acceptance and exposure techniques (24) for reducing experiential avoidance. First, the therapist introduces the experiential avoidance as a reluctance to experience unpleasant internal experiences (e.g., feelings, sensations, memories and impulses) and external experiences (e.g., situations, peoples, places) and use of internal and external avoidant strategies. Then, in the next step, he helps the patient to recognize avoidant strategies. In the final stage of this module, the patient learns to overcome his/her experiential avoidance using guidelines on acceptance and exposure to internal and external aspects.

Module 6: Preventing Relapse

The main goal of this module is to review general therapeutic concepts and discuss the progress of therapy (24). The therapist helps the patient to identify the ways to sustain the results of therapy and predict future potential problems. The patient is encouraged to use therapeutic techniques to progress in achieving short-term and long-term goals.

*Overview of Data Analysis*

The nature of data analysis in single case research continues to be a source of controversy (63). Graphical representation and visual inspection of the data remains the predominant form of analysis. Parsonson and Baer (64) argue that the goal of single case research is to detect clear and observable effects and that graphical representation and visual inspection means only such effects will be observed. It is an appropriate form of analysis for preliminary investigations into the efficacy of novel treatments. Accordingly, session by session scores across baseline, treatment and follow-up on a range of self-report measures are presented. Furthermore, pre, post and follow-up scores on outcome measures are plotted, coupled with the percentage improvement made by each patient on each measure.

Clinically Significant across (Co-) Principal Diagnoses

The clinical significance of changes during treatment should be established in order to provide a meaningful benchmark of outcome. Jacobson and colleagues (65, 66) criteria for determining reliable clinical improvement and recovery were used to determine the proportions of patients meeting these criteria at post-treatment and follow-up. This criterion requires the individual’s score at post-treatment to move from outside the range of a clinical group to within the range of a ‘functional’ group by crossing a calculated ‘cutoff point’ and to demonstrate a statistically reliable change. In that study, according to (Co-) Principal (OCD, GAD and Dysthymic), standardized criteria were developed on the BDI (criterion a; cut-off point =13, reliable change index = 7 points) for Dysthymic, the Y-BOCS (criterion a; cut-off point-14, reliable change index = 10 points) for OCD and the PSWQ (criterion a; cut-off point = 47, reliable change index = 7 points) for GAD and were used to allocate patients with (Co-) Principal to one of four possible treatment outcomes: deteriorated, unchanged, improved and recovered.

*Asymptomatic Status across (Co-) Principal Diagnoses*

An alternative, but more stringent criterion for defining recovery is that patients are asymptomatic following treatment. In this study, the defined recovery on the Y-BOCS (67), PSWQ (68) and BDI-II (38) was as a score of 7, 14 and 7 or less, respectively. This criterion was applied to each patient at the post-treatment and follow-up time points.

## Results

Demographical characteristics and clinical symptoms of patients are presented in Table 1.

**Fig 1:**
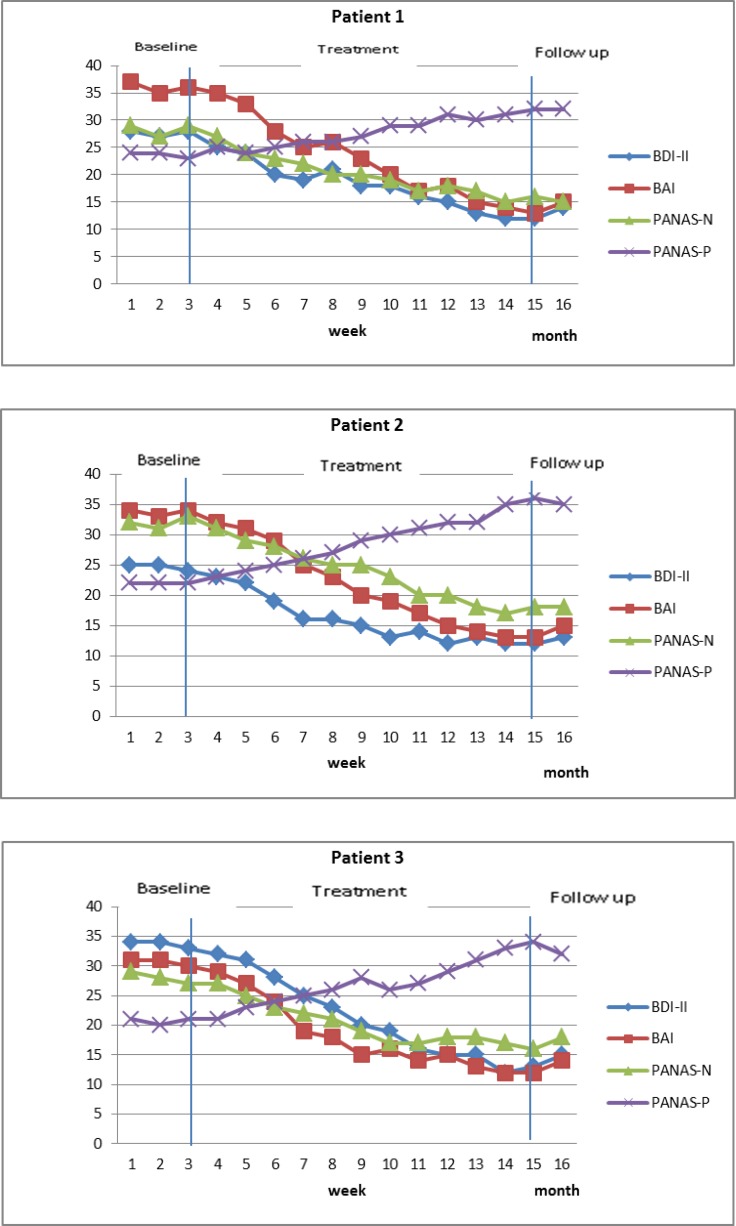
Scores on the Positive and Negative Affect Scale-Positive and Negative subscales, Beck Depression Inventory and the Beck Anxiety Inventory for Patients during Baseline, Treatment and Follow-up

**Fig 2:**
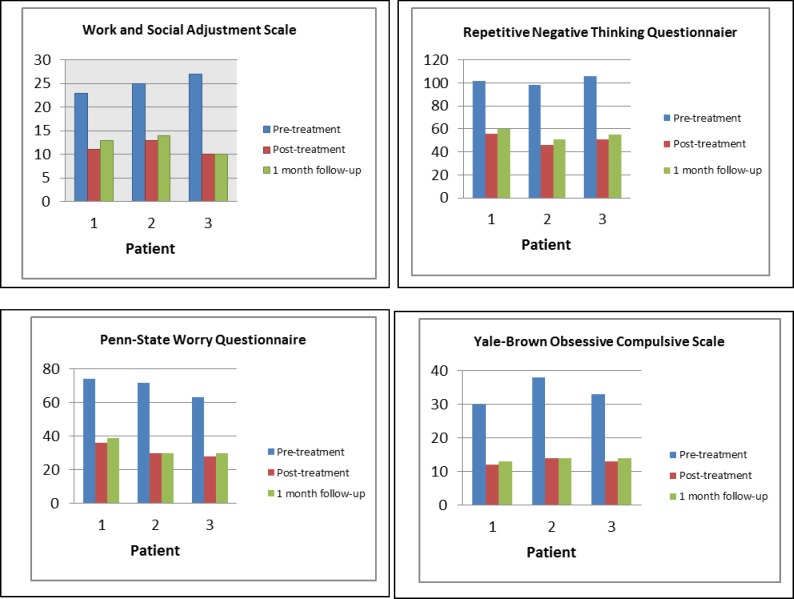
Scores on Standardized Measures at Pre-Treatment, Post-Treatment and Follow-Up for Each Patient

The distribution of mean baseline, end of therapy scores, one-month follow-up scores, baseline to end of the therapy change scores and baseline to one-month follow-up change scores were markedly skewed for the majority of the data. Therefore, the effect sizes were calculated for end of the therapy and one-month follow-up (s Table 2). Effect sizes (Cohen’s d) were calculated by dividing the mean change in individual scores (from baseline to end of therapy or one-month follow-up) by the pooled standard deviation (SD) of scores at these time-points. The pooled standard deviation is calculated as√[(SDpre^2+SDpost^2 )/2], where ‘pre’ refers to mean baseline scores and ‘post’ to end of therapy or follow-up scores. This procedure was set out by Cohen (69) and has been used and described in other case series (70-72). Cohen (68) identified effect sizes as small (d = 0.2), medium (d = 0.5) and large (0.8). Graphs were constructed for the weekly sessions and at the time-points (baseline, end of therapy, one-month follow-up) outcome measures to demonstrate detailed changes across the whole course of therapy (Figs 1 and 2).

Each patient’s scores on the BDI, the BAI and the PANAS-P, PANAS-N during the baseline and treatment phases and at follow-up are shown in Figs. 1 and 2. The baseline scores for each patient were relatively stable across all the outcome measures. Each patient showed substantial and relatively rapid reductions on the BAI, BDI and PANAS-P, PANAS-N over the course of treatment, and these reductions were largely maintained at follow-up. There was a slight deterioration at follow-up, but scores continued to represent a substantial reduction from baseline. The total score on those measures at pre, post and follow-up are illustrated in Table 2. The three patients’ pre-treatment, post-treatment and follow-up scores on the WSAS, PSWQ, Y-BOCS and RNTQ are illustrated in Fig. 2, and the total score on those measures are shown in Table 2. It can be seen that for each patient, post-treatment and follow-up scores are substantially lower than pre-treatment on all measures.

Clinical Significant across (Co-) Principal Diagnoses

At post-treatment and at one-month follow-up, all three patients met the standardized recovery criteria for principal and co-principal diagnoses on BDI-II (criterion a; cut-off point = 13, reliable change index = 7 points), the Y-BOCS (criterion a; cut-off point-14, reliable change index = 10 points) and the PSWQ (criterion a; cut-off point = 47, reliable change index = 7 points). One month follow-up data is available for the three participants who maintained recovery. The effect size estimates for the ADIS CSR’ (Co-) Principal diagnoses for all patients were in the very large range. It can be observed that the patients moved from the outside range of a “clinical group” to within the range of a “functional” group.

Asymptomatic Status across (Co-) Principal Diagnoses

When the more stringent outcome criterion of asymptomatic status for principal and co-principal diagnoses (the score of 7, 14 and 7 or less on Y-BOCS, PSWQ and BDI-II) is applied, all patients are asymptomatic at post-treatment and at one-month follow-up, both in principal and co-principal diagnoses.

*Functioning*

There were large effect sizes for an improvement in general functioning on the WSAS at end of therapy and one-month follow-up. The total mean score on the WSAS at end of therapy was also below the identified cut-off score of 10 between a clinical and general population (Mundt et al., 2002) from above this score at baseline. As shown in Fig. 2, the individual graphs showing changes on the WSAS indicate that for all participants, scores within the baseline period had been above an identified clinical mean of 25 (73), and for all participants, scores at both end of therapy and one-month follow-up were below the cut-off score of 10, specified by Mundt, Marks, Shear, and Griest (48).

**Table 1:** Demographic Characteristics, Diagnosis Conditions, Treatment History and Medication status of the Patients

**Patient**	**Age**	**Gender**	**Education**	**Marital status**	**Principal diagnosis**	**Duration of principal diagnosis**	**Co-principal diagnosis**	**Treatment history**	**Medication status**
1	22	Female	Bachelor	Single	GAD	4 year	Dysthymic OCD	No	no
2	20	Male	Bachelor	Single	OCD	7 year	GAD Dysthymic	8 sessions of CBT at 3year ago	Clomipramine 100 mg daily Fluoxetine, 20 mg daily
3	23	Female	Bachelor	Single	Dysthymic	5 year	OCD GAD	5 sessions of CBT at 4 year ago	no

Note. GAD: Generalized Anxiety Disorder; OCD: Obsession Compulsive Disorder, CBT: Cognitive Behavioral Therapy.

**Table 2:** Descriptive Statistics and Effect Sizes for Primary Outcome Variables at end of treatment and 1-month follow-up

**Measure**	**Mean baseline** **M(SD)**	**End of therapy** **M(SD)**	**Effect size** **Pre-post**	**1 month follow-up** **M(SD)**	**Effect size** **pre-1m FU**
ADIS (Co-)Principal Dx CSR	6.50 (0.6)	2.90 (0.7)	3.91	2.46 (1.08)	3.28
BDI	28.8 (4.7)	12.3 (3.9)	2.77	14 (3.2)	2.42
BAI	33.4 (5.2)	12.6 (3.7)	3.26	14.6 (3.1)	3.1
PANAS-N	29.4 (4.4)	16.6 (3.8)	2.17	17 (2.4)	2.67
PANAS-P	22.1 (3.7)	34 (4.2)	1.99	33 (4.9)	1.77
WSAS	25 (4.1)	11.3 (3.5)	2.53	12.3 (4.3)	2.13
RNTQ	106 (16.2)	43 (8.2)	3.47	45.3 (9.3)	3.25
PSWQ	69.6 (11.2)	31.3 (12.4)	2.11	33 (9.4)	2.50
Y-BOCS	33.6 (5.9)	13 (4.1)	2.86	13.6 (3.9)	2.82

M = mean; SD = standard deviation; ADIS = Anxiety Disorders Interview Schedule; CSR = clinical severity rating; BDI=Beck Depression Inventory; BAI=Beck Anxiety Inventory; PANAS-P and PANAS-N=Positive and Negative Affect Scale-Positive and Negative subscales; WSAS=Work and Social Adjustment Scale; RNTQ=Repetitive negative thinking questionnaire.

## Discussion

The fundamental goal of this study was to answer one question: Could transdiagnostic treatment based on repetitive negative thinking be effective in treating the co-occurrence of anxiety and depressive disorders? The results of this case series provide preliminary evidence for effectiveness of this protocol for treating the co-occurrence of anxiety and depressive disorders. Substantial reductions were obtained on all the main outcome measures at post-treatment and at one-month follow-up, including the predicted changes on repetitive negative thinking as the core transdiagnostic factor in emotional disorders, especially anxiety and depression disorders. Every participant achieved recovery at post-treatment and at one-month follow-up in (co-)principal diagnosis according to the standardized clinical significance criteria on the BDI-II, Y-BOCS, PSWQ and the ADIS CSR. In terms of the asymptomatic criterion, all participants were asymptomatic at post-treatment and at one-month follow-up. In addition, transdiagnostic treatment based on repetitive negative thinking appears to be relatively time efficient and easily delivered treatment for multiple disorders, achieving good treatment outcomes in 12 hours of therapy. Interestingly, the transdiagnostic treatment based on repetitive negative thinking evidenced large treatment effect for improvement in general functioning on the WSAS at end of therapy and one-month follow-up. 

Importantly, the transdiagnostic treatment based on repetitive negative thinking was effective in the treatment of a range of anxiety and depression disorders, including GAD, OCD and depression, yielding effect sizes comparable to treatments targeting disorder-specific symptoms. In this study, all patients receiving the transdiagnostic treatment based on repetitive negative thinking no longer met diagnostic criteria for their principal and co-principal diagnosis. Significantly, these patients no longer met criteria for any clinical diagnosis at post-treatment and follow-up. 

 Transdiagnostic treatments targeting core “higher-order” factors offer a more parsimonious approach to treatment planning that eliminates the need for multiple diagnosis-specific manuals (17). In addition, other researchers have begun to consider how existing evidence-based therapeutic principles could be effectively applied transdiagnostically on a more empirical basis using evidence-based modules of behavior change procedures (15- 17, 29, 74-78). Some of these efforts focus on identifying and correcting deficits in functioning rather than focusing on cross-cutting dimensions of psychopathology.

In what is perhaps the most advanced effort along these lines, Fairburn and colleagues (79) have developed a transdiagnostic approach to eating disorders based on shared psychological dimensions of these disorders, an approach similar to but predating ours (13). Regardless of the strategy, these transdiagnostic approaches may not only prove to be more effective, but also have significant implications for broader dissemination efforts. More specifically, transdiagnostic treatments have the potential to reduce the amount of time and effort that is required for adequate training, a factor that has hindered dissemination efforts in the past (80-82). Also, if proved effective, these treatments may prove to have considerable clinical utility. Clinicians are often faced with the difficult task of treating individuals with complex clinical presentations that require them to use multiple protocols or to tackle several problems at once, with little empirical data to guide them. Transdiagnostic treatments may help eliminate the need for multiple diagnosis-specific treatment manuals and simplified treatment planning.

## Limitation

There are a number of limitations of this case series. First, the generalizability of the results is limited as only three patients were treated. However, they were seen in clinical practice as no one was excluded on the basis of comorbid disorders as it is often the case in randomized controlled trials.

 A second limitation is that outcome assessment relied heavily on self-report measures and the therapist administered the only clinician rated measure, the Y-BOCS.

Third, treatment was delivered by only one therapist that may limit the generalizability of the results; replication with other therapists is required, but this was not possible in this design as it was a replication across patients. However, treatment was delivered by a relatively inexperienced cognitive therapist which may be a further indication of the effectiveness of transdiagnostic treatment based on repetitive negative thinking.

Forth, this study had a large number of outcome measures. Due to the small sample size, it is acknowledged that statistically this can increase the chances of error. The small sample size also limits the degree to which the results are generalizable to a ‘typical’ population. Nevertheless, the present results suggest that continuing evaluations of this new treatment approach are warranted. 

Thus, due to these limitations, the observed improvements cannot be unequivocally attributed to TTRNT, recommending that the future studies apply transdiagnostic interventions in the selective and comprehensive randomized controlled trial research. Furthermore, a long-term follow up for outcomes of transdiagnostic treatment based on repetitive negative thinking is suggested. The study of transdiagnostic therapy mechanism and its efficacy in the other disorders and problems is necessary for the procedure dismantling studies. Finally, it seems that more studies need to be conducted on transdiagnostic interventions to compare specific interventions and the other disorders with this approach.

## Conclusion

In conclusion, comorbidity of anxiety and depression disorders is a big challenge in specific cognitive-behavioral therapies and transdiagnostic therapies are one of the solutions. Effectiveness of these protocols has been evolving and the breakthrough was the Barlow’s transdiagnostic protocol, which was a great step in empowering these therapies by reinforcing the theoretical foundation of pragmatic protocols. However, these therapies still have moderate effectiveness in the best condition. Reviewing theoretical studies, this shortage can be attributed to ignoring the main process in emotional disorders (i.e., repetitive negative thinking) and its relevant variables. In summary, all the three patients treated with TTRNT made clinically significant improvement within 12 hours of therapy. TTRNT for co-occurrence of anxiety and depressive disorders could prove to be a time effective and efficient treatment for individuals with these occurrence disorders. The results of this preliminary investigation revealed that a controlled evaluation of the efficacy of TTRNT for co-occurrence of anxiety and depressive disorders is needed. An appropriate next step would be to conduct a randomized controlled trial comparing TTRNT and Barlow’s transdiagnostic protocol or a well-established cognitive behavioral treatment. To this end, we are conducting a pilot randomized controlled trial of TTRNT for co-occurrence of anxiety and depressive disorders, using the treatment manual from this study to more rigorously investigate the efficacy of TTRNT as a new form of transdiagnostic treatment.
